# Case report: Cyclophosphamide pulse therapy for chronic recalcitrant erythema nodosum leprosum

**DOI:** 10.3389/fmed.2023.1272404

**Published:** 2023-10-25

**Authors:** Gustavo U. Machado, Thiago Amparo, Flávia Bulhões, Paulo R. L. Machado

**Affiliations:** ^1^Serviço de Imunologia, Hospital Universitário Prof. Edgar Santos, Universidade Federal da Bahia, Salvador, Brazil; ^2^Serviço de Dermatologia, Hospital Universitário Prof. Edgar Santos, Universidade Federal da Bahia, Salvador, Brazil; ^3^Instituto Nacional de Ciência e Tecnologia em Doenças Tropicais, Salvador, Brazil

**Keywords:** leprosy, pulse therapy, cyclophosphamide, erythema nodosum leprosum (ENL), type 2 reaction

## Abstract

Chronic recalcitrant erythema nodosum leprosum (ENL) or type 2 reaction (T2R) is a severe condition found in approximately 50% of multibacillary leprosy subjects. T2R is associated with important morbidities and may lead to several disabilities, not only due to nerve damage but also due to the prolonged use of corticosteroids, thalidomide, or immunosuppressors. We describe here four leprosy patients with chronic recalcitrant ENL treated with cyclophosphamide pulse therapy. All subjects had been on prednisone and thalidomide therapy for at least 30 months but showed inflammatory activity when doses were reduced. Pulse therapy with 1.0 g of cyclophosphamide was used every 4–6 weeks for a minimum of three applications. After pulse therapy, all cases presented total or partial regression of symptoms, and we were able to taper thalidomide and prednisone doses, with better control of ENL, avoiding further hospital admissions and disabilities. No side effects were observed during or after infusion therapy. Cyclophosphamide pulse therapy may be useful and safe to control chronic recalcitrant ENL.

## Introduction

Erythema nodosum leprosum (ENL) or type 2 reaction (T2R) is a common and severe immune-inflammatory complication of multibacillary leprosy. T2R is associated with increased levels of inflammatory cytokines and chemokines, such as TNF and IL-6, among others, not only in cutaneous lesions but also in several internal organs, leading to systemic involvement. ENL is characterized by the presence of subcutaneous acute and painful nodules associated with fever, myalgia, asthenia, arthritis, neuritis, generalized lymphadenopathy, and many other symptoms, sometimes requiring hospitalization (1, 2).

Chronic recalcitrant ENL is a hard-to-treat condition that imposes long-term use of corticosteroids, thalidomide, or immunosuppressors, resulting in significant morbidity and increasing the risk of disability in those patients (1, 2). Other drugs, such as pentoxifylline and clofazimine, have been used but seem to be effective in less severe cases. More recently, other therapeutic strategies have been used in an attempt to control ENL, such as anti-TNFα agents and apremilast (3–5).

Pulse therapy with cyclophosphamide has been advocated for the treatment of connective tissue diseases, neutrophilic dermatosis, bullous diseases, and other autoimmune and inflammatory conditions (6).

In this study, we describe four leprosy patients with chronic recalcitrant ENL who were under prednisone and thalidomide therapy for at least 30 months. Pulse therapy with 1.0 g of cyclophosphamide was used every 4–6 weeks for a minimum of three applications.

## Case description

From January 2018 to July 2023, four ENL patients were evaluated after inclusion in the cyclophosphamide pulse therapy protocol due to chronic, relapsing, and difficult-to-control episodes of severe ENL after at least three attempts to lower the dose after taking prednisone or thalidomide. Table 1 shows demographic, clinical, and therapeutic characteristics. The females predominate males at a 3:1 ratio, with ages ranging from 24 to 41 years. Patients were diagnosed using the Ridley–Jopling criteria (7) upon clinical evaluation, histopathology, and a positive bacillary index. Serology tests for HIV, HTLV-1, B, and C hepatitis viruses were negative (8). All cases had long-term use (36–61 months) of prednisone or thalidomide. Prednisone daily doses ranged from 80 to 2.5 mg, and thalidomide daily use varied from 400 to 50 mg.

**Table 1:** Demographic, clinical and therapeutic characteristics of chronic relapsing ENL patients.

Patient	Age	Sex	R&J	Duration of ENL (months)	ENL characteristics	Pulse therapy cycles	Thalidomide before pulse therapy*	Thalidomide after pulse therapy**	Prednisone before pulse therapy*	Prednisone after pulse therapy**	Degree of disability before	Degree of disability after	Clinical outcome
1	41	F	LL	62	Subcutaneous nodules, acroedema, arthralgia, fever, lymphadenopathy, neuritis	6	200 mg	200 mg	60 mg	10 mg	2	2	Few and small nodules; no other symptoms; using thalidomide 200 mg and prednisone 10 mg
2	36	F	BL	58	Subcutaneous nodules, fever, myalgia	5	400 mg	100 mg	50 mg	18 mg	0	0	Discharge after 5 months without drugs and no clinical activity
3	28	M	LL	48	Subcutaneous nodules, acroedema, arthritis^¶^, fever, myalgia	4	400 mg	400 mg	60 mg	10 mg	0	0	Polyarthralgia, no nodules or other symptoms; thalidomide 400 mg and prednisone 5 mg; methotrexate 20 mg weekly (using before pulse therapy)
4	24	F	LL	55	Subcutaneous nodules, acroedema, arthralgia, astenia, fever, headache	3	400 mg	400 mg	20 mg	20 mg	0	0	No nodules or other symptoms; thalidomide 400 mg; prednisone 15 mg

*Daily dosage in average from the first ENL episode until the first pulse therapy infusion. **Daily dosage in average from the last pulse therapy until the last consultation. ^¶^A seronegative polyarthritis since the first ENL episode, requiring methotrexate use (20 mg/week) associated with prednisone and thalidomide before pulse therapy.

The duration of ENL ranged from 48 to 62 months, and all cases were classified as presenting a severe reaction (9) with several episodes of reactivation during the observation period, especially upon any tentative lower prednisone or thalidomide doses. During the clinical activity of T2R, the patients presented more than 20 subcutaneous nodules associated with systemic symptoms such as fever, myalgia, arthritis, neuritis, lymphadenopathy, and edema of the extremities (Table 1). Three out of four subjects were presented with ENL before multidrug therapy (MDT), whereas one patient (number 2) developed ENL during MDT. However, this patient was diagnosed with a reversal reaction (RR) before MDT. She presented BL, and after this first RR and MDT initiation, she developed a series of recurrent ENL episodes, and no further RR was detected.

Cyclophosphamide pulse therapy was initiated during hospitalization for 1 or 2 days with 1.0 g diluted in saline 0.9% by intravenous infusion in 4 h. Before pulse therapy, all subjects performed the following laboratory evaluations: blood count, liver enzymes, blood glucose, BUN, creatinine, chest x-ray, and urinalysis. No adverse events (AEs) were associated with pulse therapy, in contrast with several AEs presented due to prednisone and thalidomide chronic use, such as Cushing syndrome, acne, diabetes, and deep venous thrombosis. After more than 3 years of prednisone and thalidomide use, one patient (number 4) was diagnosed with latent tuberculosis. She was treated with isoniazid and rifampicin and considered cured.

A positive effect of cyclophosphamide pulse therapy in all patients was confirmed by a better control of ENL symptoms, allowing the use of a lower dosage of prednisone or thalidomide (Table 1). The daily average dosage of thalidomide dropped in only one patient, from 400 to 100 mg. However, the prednisone daily average dose was lower in three out of four subjects (50–74% reduction) after pulse therapy.

In the three subjects presenting no disabilities at diagnosis and before ENL treatment, despite the chronicity and severity of reaction episodes, it was possible to avoid any development of disabilities. Patients 2, 3, and 4 needed to be hospitalized due to the intensity of their reaction before using pulse therapy. No one needs hospitalization due to ENL symptoms after the first cyclophosphamide cycle. One subject (number 2) was discharged from the outpatient clinics after no signs or symptoms of ENL without using prednisone, thalidomide, or any other immunosuppressive drug for at least 5 months. However, the other three patients remain on low-dose prednisone and thalidomide therapy.

## Discussion

Reactions are the main source of disabilities in leprosy, not only associated with neuritis but also with systemic involvement. ENL may be a longstanding complication of leprosy in approximately 50% of multibacillary cases, leading to the use of high dosages of thalidomide, prednisone, or immunosuppressors for a long period of time, in most cases, for many years (1, 2). Unfortunately, most patients develop severe morbidities and even death associated with prolonged use of corticosteroids (2). In addition to all the physical consequences, the impact of reactions in social, economic, and psychological domains may be underestimated (10–12). Leprosy remains a burden in more than 120 countries and is considered by the WHO to be the most common infectious cause of disability in the world (13). Nevertheless, the incredible negligence toward the disease is reflected in the very few alternatives and trials for the development of new drugs that are more effective and safer for managing reactions (14).

ENL is mediated by increased peripheral production of chemokines and cytokines like IL-6, IFN, IL-17, and TNFα, immune complex deposits, and neutrophil infiltration in the skin and internal organs. There is also the participation of T-cells and the activation of intermediate monocytes, which contribute to the development of tissue damage (15–17).

In addition to corticosteroids and thalidomide, which prolonged use is associated with several side effects, other options such as pentoxifylline, clofazimine, and immunosuppressors may also require a long period of use, with variable effectiveness along with toxicity. More recently, anti-TNFα drugs and apremilast have been used for treating chronic and difficult-to-control ENL with favorable results. Etanercept (6 cases), infliximab (2 cases), and adalimumab (1 case) were employed in variable dosages, and rapid response (hours) was observed with infliximab use (3, 4, 18). However, no prospective controlled trial has been published yet, and besides the high costs, anti-TNFα agents may be associated with the reactivation of tuberculosis. Additionally, anti-TNFα therapy has been associated with leprosy relapse or the efficacy of MDT drugs in leprosy patients under treatment (4). In a pilot study, apremilast—an oral phosphodiesterase-4 inhibitor that decreases the Il-17 pathway and multiple inflammatory cytokines—was used in 12 patients with chronic or recurrent ENL for 6 months with promising results (5). Unfortunately, apremilast has a high cost and may require prolonged use, limiting its indication.

Due to its immunosuppressive effects, cyclophosphamide is used as a treatment for various autoimmune diseases. It suppresses T and B cells and decreases antibodies, adhesion molecules, and cytokine production (6, 19). Cyclophosphamide has shown a role in corticosteroid-sparing in pemphigus and lupus disease (20–22). In the treatment of autoimmune diseases, intravenous cyclophosphamide pulse therapy has been administered at 500–1,000 mg/m^2^, at 3–4 weeks for 3–6 months, alone or in association with methylprednisolone (6, 20, 21). Although daily oral administration is possible, pulse therapy has been shown to be safer (less leukopenia, amenorrhea, and teratogenicity) without difference in reactivation rates for vasculitic diseases (22, 23).

Our data suggest that pulse therapy with cyclophosphamide may be useful to avoid high dosages of prednisone, hospitalizations due to severe ENL relapses, and the development of disabilities. All these advantages may also provide a potential cost-effectiveness advantage in favor of pulse therapy use. The limitations of our case series are the retrospective design and the absence of a control group. Additionally, the cyclophosphamide pulse therapy schedule was used for variable periods of time ranging from 4 to more than 6 weeks, which could not be enough to achieve the necessary immune-inflammatory modulation required for a better therapeutic outcome. However, pulse therapy with cyclophosphamide should be considered in steroid-dependent patients with severe and recrudescent ENL. To our knowledge, there is no previous data about the use of cyclophosphamide pulse therapy in the management of ENL. Future prospective and controlled studies in a larger number of patients should be conducted to evaluate the efficacy of cyclophosphamide pulse therapy in the treatment of chronic recalcitrant ENL.

## Data availability statement

The raw data supporting the conclusions of this article will be made available by the authors, without undue reservation.

## Ethics statement

The studies involving humans were approved by the Fameb, Federal University of Bahia. The studies were conducted in accordance with the local legislation and institutional requirements. The participants provided their written informed consent to participate in this study. Written informed consent was obtained from the individual(s) for the publication of any potentially identifiable images or data included in this article. Written informed consent was obtained from the participant/patient(s) for the publication of this case report.

## Author contributions

GM: Data curation, Formal analysis, Investigation, Methodology, Writing – original draft, Writing – review & editing, Validation. TA: Data curation, Investigation, Methodology, Writing – original draft, Validation. FB: Data curation, Methodology, Writing – original draft. PM: Conceptualization, Data curation, Formal analysis, Funding acquisition, Investigation, Methodology, Supervision, Writing – original draft, Writing – review & editing, Validation.
